# Three-round learning strategy based on 3D deep convolutional GANs for Alzheimer’s disease staging

**DOI:** 10.1038/s41598-023-33055-9

**Published:** 2023-04-07

**Authors:** Wenjie Kang, Lan Lin, Shen Sun, Shuicai Wu

**Affiliations:** Beijing International Platform for Scientific and Technological Cooperation, Department of Biomedical Engineering, Faculty of Environment and Life Sciences, Beijing University of Technology, Beijing, 100124 China

**Keywords:** Alzheimer's disease, Biomedical engineering, Neuroscience

## Abstract

Accurately diagnosing of Alzheimer's disease (AD) and its early stages is critical for prompt treatment or potential intervention to delay the the disease’s progression. Convolutional neural networks (CNNs) models have shown promising results in structural MRI (sMRI)-based diagnosis, but their performance, particularly for 3D models, is constrained by the lack of labeled training samples. To address the overfitting problem brought on by the insufficient training sample size, we propose a three-round learning strategy that combines transfer learning with generative adversarial learning. In the first round, a 3D Deep Convolutional Generative Adversarial Networks (DCGAN) model was trained with all available sMRI data to learn the common feature of sMRI through unsupervised generative adversarial learning. The second round involved transferring and fine-tuning, and the pre-trained discriminator (D) of the DCGAN learned more specific features for the classification task between AD and cognitively normal (CN). In the final round, the weights learned in the AD versus CN classification task were transferred to the MCI diagnosis. By highlighting brain regions with high prediction weights using 3D Grad-CAM, we further enhanced the model's interpretability. The proposed model achieved accuracies of 92.8%, 78.1%, and 76.4% in the classifications of AD versus CN, AD versus MCI, and MCI versus CN, respectively. The experimental results show that our proposed model avoids overfitting brought on by a paucity of sMRI data and enables the early detection of AD.

## Introduction

Dementia is a leading cause of disability in people over 65 years old worldwide^1,2^. Alzheimer’s disease (AD) is the principal cause of dementia, characterized by memory loss and cognitive decline caused by the deposition of amyloid-β protein^3,4^. According to AD International^5^, at least 50 million people worldwide are affected by AD, and this number is expected to triple to 152 million by 2050^6^. Over the last decade, there has been a growing interest in the diagnosis of AD and its preclinical state, mild cognitive impairment (MCI) (a transitional state between CN and AD). Structural magnetic resonance imaging (sMRI) is a non-invasive neuroimaging technology for measuring neural damage and disease progression that has been used in the computer-aided diagnosis of AD and/or MCI.

In recent years, deep learning methods have shown great capacity for many computer vision tasks^7,8^. A typical deep learning model, convolutional neural network (CNN), has been widely used in the neuroimaging community, especially in AD classification^9^. Neuroimaging studies usually have a limited amount of data. To address this problem, a lot of scientific research on AD classification^10–12^ sliced 3D brain volumes into two dimensional (2D) images, adopted a classical 2D CNN pre-trained by natural images as a starting point, and fine-tuned the network through transfer learning. Nanni et al.^13^ applied the pre-trained AlexNet, GoogleNet, ResNet50, ResNet101, and InceptionV3 to sMRI, and obtained the area under the receiver operating characteristic curve (AUC) of 90.8%, 89.6%, 89.8%, 89.9%, and 88.8%, respectively, when comparing AD and CN. In our previous work^14^, we improved the performance of 2D CNNs by utilizing multi-slice and multi-model integration. However, these 2D-based approaches ignore the spatial information between slices, making it impossible to fully utilize 3D contextual data.

Compared with a 2D CNN, a 3D CNN can utilize richer spatial 3D contextual information and generate more discriminative features. 3D CNN has been applied to the staging of the AD spectrum. Kong and his colleagues^15^ initially trained a 3D sparse autoencoder to learn the filters on randomly chosen 3D patches of the sMRI and then used those pretrained kernels as the first convolution layer of a 3D CNN. Li et al.^16^ proposed a hybrid convolutional and recurrent neural network by combining 3D DenseNets and (bidirectional gated recurrent unit) BGRU for AD diagnosis based on hippocampus volumes. 3D DenseNet was then utilized to learn the various local features from image patches, while BGRU was applied to capture the high-level correlation features between the left and right hippocampus. In Li et al.^17^’s work, three 3D VGG-like CNNs were applied to capture the features of the 3D hippocampal shapes and asymmetry. The cascaded 2D CNN learned the high-level correlation features between two hippocampi, and the features learned by the asymmetry channel and 2D CNNs were combined with a fully connected layer. Liu et al.^18^ constructed a multi-task deep CNN model for jointly learning hippocampus segmentation and AD classification. The features from 3D U-Net and DenseNet were combined for AD classification. Huang et al.^19^ proposed a hybrid 3D VGG + support vector machine (SVM) model in which CNN was used to extract features and the SVM was used to obtain classification results based on the extracted features. The model consisted of three branches; each branch was for a binary classification, and three branches were fused for a ternary classification.

Convolutional layers have trainable parameters that are independent of image size. However, the number of trainable parameters in the subsequent fully connected layers depends on the size of the feature map of the last convolutional layer. In 2D CNNs, this is not an issue because convolutional filters learn smaller latent representations from 2D images. However, in 3D CNNs, the latent representations of the last convolutional layer grow in size, increasing the size of the weights that need to be learned in the first fully connected layers and making training more difficult. Despite the fact that 3D CNNs can solve the problem of discontinuity across slices, extending 2D CNNs to 3D CNNs faces significant challenges, such as high memory and computational costs, the curse of dimensionality for high-dimensional data, and the phenomenon of overfitting. 3D CNNs might require a larger training dataset than their 2D counterparts due to the larger number of parameters. Therefore, preventing the overfitting phenomenon during the training process caused by the data scarcity is very important. A possible solution is cross-domain transfer learning. Liu et al.^20^ aggregated the training dataset from several medical challenges with diverse modalities, target organs, and pathologies and trained a 3D segmentation network. The residual CNN pre-trained in the hippocampal segmentation task was then transferred for AD versus CN classification. However, this strategy has not yet yielded the expected results because of the large differences between different modalities, target organs, and pathologies of medical images.

Generative adversarial networks (GAN)^21^ is an unsupervised deep learning model based on the idea of a zero-sum game. It includes two competing networks: a generative network (G) and a discriminant network (D). The adversarial game between G and D allows the G to generate convincing samples, and the D to have a better feature extraction capability. G and D are pitted against each other and eventually reach a Nash equilibrium. The GAN model defines adversarial goals between the G and the D, and allows the D to better learn the common features of the training images through adversarial learning and feature matching. This could provide an attractive solution to overfitting in 3D CNNs by first using the D network as a common feature extractor and then reusing the D network as a starting point for supervised training. The main contributions of this work are outlined as follows:

A) We extended 2D Deep Convolutional GANs (DCGAN) into 3D DCGAN and integrated the residual block concept into DCGAN to improve the feature extraction ability.
B) A three-round learning strategy (unsupervised adversarial learning for pre-training a classifier and two-round transfer learning for fine-tuning the classifier)is proposed to solve the problem of overfitting in 3D CNNs caused by small samples and improve the classification performance in AD staging. To the best of our knowledge, we first introduced the 3D DCGAN in AD classification.
C) Grad-CAM visualizations are integrated into the system and provide useful explanations of its predictions.

The rest of the paper is organized as follows: Section "Material and methods" introduces the data preprocessing procedures, the three-round training procedure, and a diagnosis model based on a 3D DCGAN. Section "Experiment and results" presents the experimental settings and experimental results of this research. Section "Discussion" discusses the overall results and possibilities for future work, and Section "Conclusions" concludes the study.

## Material and methods

### Dataset

Data used in the preparation of this article were obtained from the ADNI. The ADNI was launched in 2003 as a public–private partnership, led by Principal Investigator Michael W. Weiner, MD. The primary goal of ADNI has been to test whether MRI, positron emission tomography, other biological markers, and clinical and neuropsychological assessment can be combined to measure the progression of MCI and AD^22,23^. All relevant tests and methods were performed in accordance with relevant guidelines and regulations, and this study was approved by the ADNI Publications Committee.

1.5 T T1-weighted baseline sMRI scans from 798 participants of the ADNI-1 cohort were included in this study (187 AD, 382 MCI, and 229 CN). The MMSE scores were significantly different between the AD, MCI, and CN groups. Table 1 shows the demographic characteristics of the three groups of participants. MCI subjects were further divided into two subgroups: progressive MCI (pMCI) and stable MCI (sMCI), according to 24-months cognitive assays. Some MCI patients were excluded from further analysis due to incomplete follow-up conversion status, or reversion status. Thus, of the 382 MCI subjects, 138 pMCI and 181 sMCI participants remained in the study of the prediction of MCI converting to AD. The entire data set was randomly split into training, validation, and test sets in a ratio of 7: 1: 2.

**Table 1:** Demographic information of the subjects in ADNI-1.

Characteristic	AD	MCI	CN
Subjects	187	382	229
Age	75.26 ± 7.53	74.71 ± 7.48	75.87 ± 5.02
Gender (male/female)	98/89	245/137	119/110
Education	14.66 ± 3.14	15.67 ± 2.90	16.07 ± 2.85
Mini-mental state examination scores (MMSE)	23.28 ± 2.04	27.33 ± 1.83	29.11 ± 1.00

The age, education years, and MMSE values are reported as Mean ± Standard deviation (Std).

### Image pre-processing

The anatomical MRI scans were reconstructed from the Digital Imaging and Communications in Medicine (DICOM) file and converted to the Neuroimaging Informatics Technology Initiative (NIfTI) format, using dcm2niigui (distributed by MRIcron). Image pre-processing was performed with the SPM12 (http://www.fil.ion.ucl.ac.uk/spm/software/spm) toolbox Computational Anatomy Toolbox 12 (CAT12, http://www.neuro.uni-jena.de/cat/)**.** T1 images were first bias-field inhomogeneity corrected, registered using an initial affine transformation, followed by non-linear deformation. Then, the normalized images were segmented into gray matter (GM), white matter, and cerebrospinal fluid tissue classes and modulated. Before being used for further analysis, the extracted GM density maps (GMDM) were smoothed with a 2.0 mm full width at half maximum (FWHM) Gaussian isotropic kernel. The preprocessed GMDM had the dimension of 121 × 145 × 121 with an isotropic resolution of 1.5 mm. Finally, due to GPU memory limitations, the GMDM were cropped and padded to 128 × 128 × 128 voxels and down sampled to 64 × 64 × 64 voxels with an isotropic resolution of 3.0 mm.

### 3D DCGAN architecture

**Figure 1:**
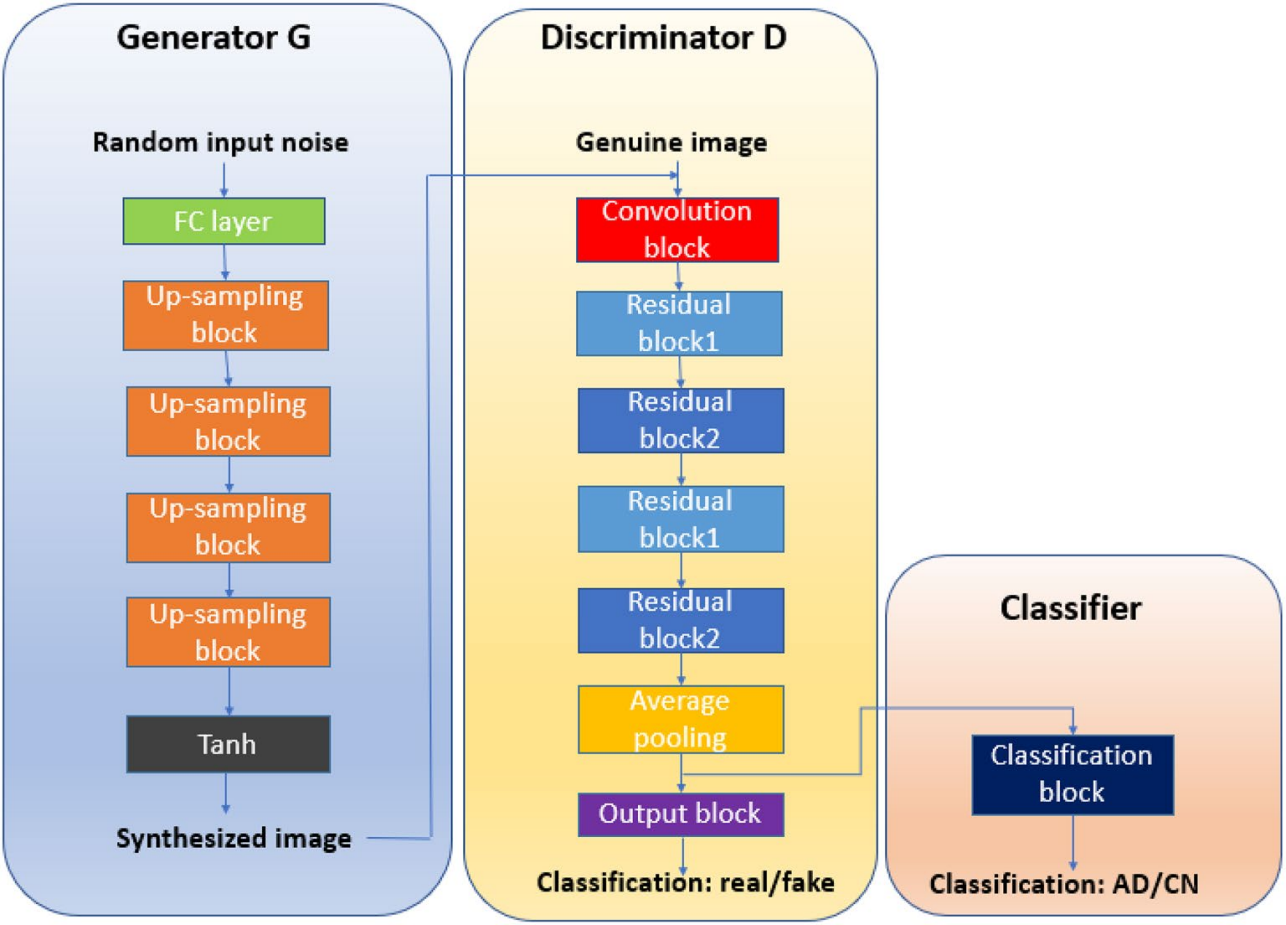
Flowchart of the proposed 3D DCGAN.

An important characteristic of GANs is unsupervised representation extraction from unlabeled data. DCGAN^24^ is a milestone improvement of the original GAN by building the GAN structure with CNNs. In this work, we have proposed a 3D version of the DCGAN, where D uses four residual blocks to improve the feature representations with low memory usage. In the DCGAN, the G synthesizes T1 weighted sMRI of the whole brain, and the D discriminates between genuine sMRI and synthetic sMRI. Two networks are constantly competing against each other. Competition between the G and D through an adversarial process helps both networks learn the statistical distribution of unlabeled neuroimaging data. When the network reached Nash equilibrium, a two-round transfer learning strategy was applied. The first round of transfer learning is used for AD classification, and the second round of transfer learning is applied for other binary tasks. The flowchart of the 3D DCGAN is shown in Fig. 1.

**Figure 2:**
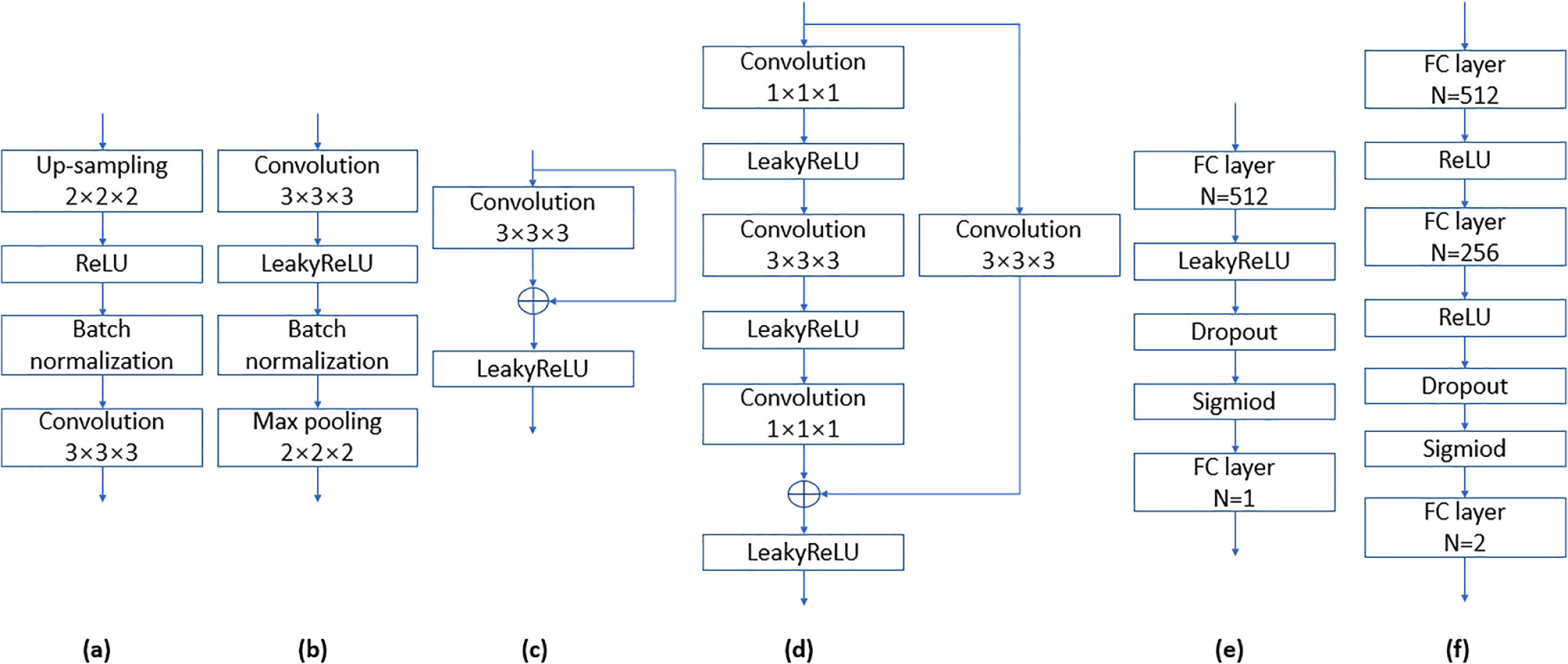
The network architecture of (**a**) up-sampling block (**b**) convolution block (**c**) residual block1 (**d**) residual block2 (**e**) output block (**f**) classification block.

G takes a latent vector of size 100 drawn from a normal Gaussian distribution as input. The G network yields a synthetic sMRI from random vectors that fits into the genuine sMRI distribution by utilizing up-sampling blocks. The D network discriminates if the input is a genuine sMRI or whether it’s a synthetic sMRI generated by the G network. As for the network architecture of D, we constructed a 3D ResNet-like CNNs by presenting shortcut connections, which can stimulate the training speed and overcome the impact of the vanishing gradient problem. D consists of a convolution block, four residual blocks, and an output block. The residual blocks in D include two different architectures. Residual block1 and block3 are the standard residual blocks, and residual block2 and block4 are bottleneck blocks. The bottleneck structure reduces the amount of calculation by adding a 1 × 1 × 1 convolution layer to the standard residual module to reduce the number of features. A dropout layer was set in the output block to alleviate overfitting. The detailed architectures of G and D are demonstrated in Fig. 2, Tables 2 and 3.

**Table 2:** The network architecture of G.

Layers		Filter size, number, stride	Output size
Input layer		–	100
FC layer		–	4 × 4 × 4 × 128
Up-sampling block		3 × 3 × 3, 64, 1	8 × 8 × 8, 64
Up-sampling block		3 × 3 × 3, 32, 1	16 × 16 × 16, 32
Up-sampling block		3 × 3 × 3, 16, 1	32 × 32 × 32, 16
Up-sampling block		3 × 3 × 3, 1, 1	64 × 64 × 64, 1

**Table 3:** The network architecture of D.

Layers	Filter size, number, stride	Output size
Input layer	–	64 × 64 × 64, 1
Convolution block	3 × 3 × 3, 16, 1	64 × 64 × 64, 16
Max pooling	2 × 2 × 2, 16, 2	32 × 32 × 32, 16
Residual block1	3 × 3 × 3, 16, 1	32 × 32 × 32, 16
Residual block2	$$\left[\begin{array}{c}1\times 1\times \mathrm{1,32,1}\\ 3\times 3\times \mathrm{3,32,2}\\ 1\times 1\times \mathrm{1,32,1}\end{array}\right]$$	16 × 16 × 16, 32
Residual block3	3 × 3 × 3, 32, 1	16 × 16 × 16, 32
Residual block4	$$\left[\begin{array}{c}1\times 1\times \mathrm{1,64,1}\\ 3\times 3\times \mathrm{3,64,2}\\ 1\times 1\times \mathrm{1,64,1}\end{array}\right]$$	8 × 8 × 8, 64
Average pooling	2 × 2 × 2, 64, 2	4 × 4 × 4, 64
Output block	$$\left[\begin{array}{c}512\\ 1\end{array}\right]$$	1

### Transfer learning

**Figure 3:**
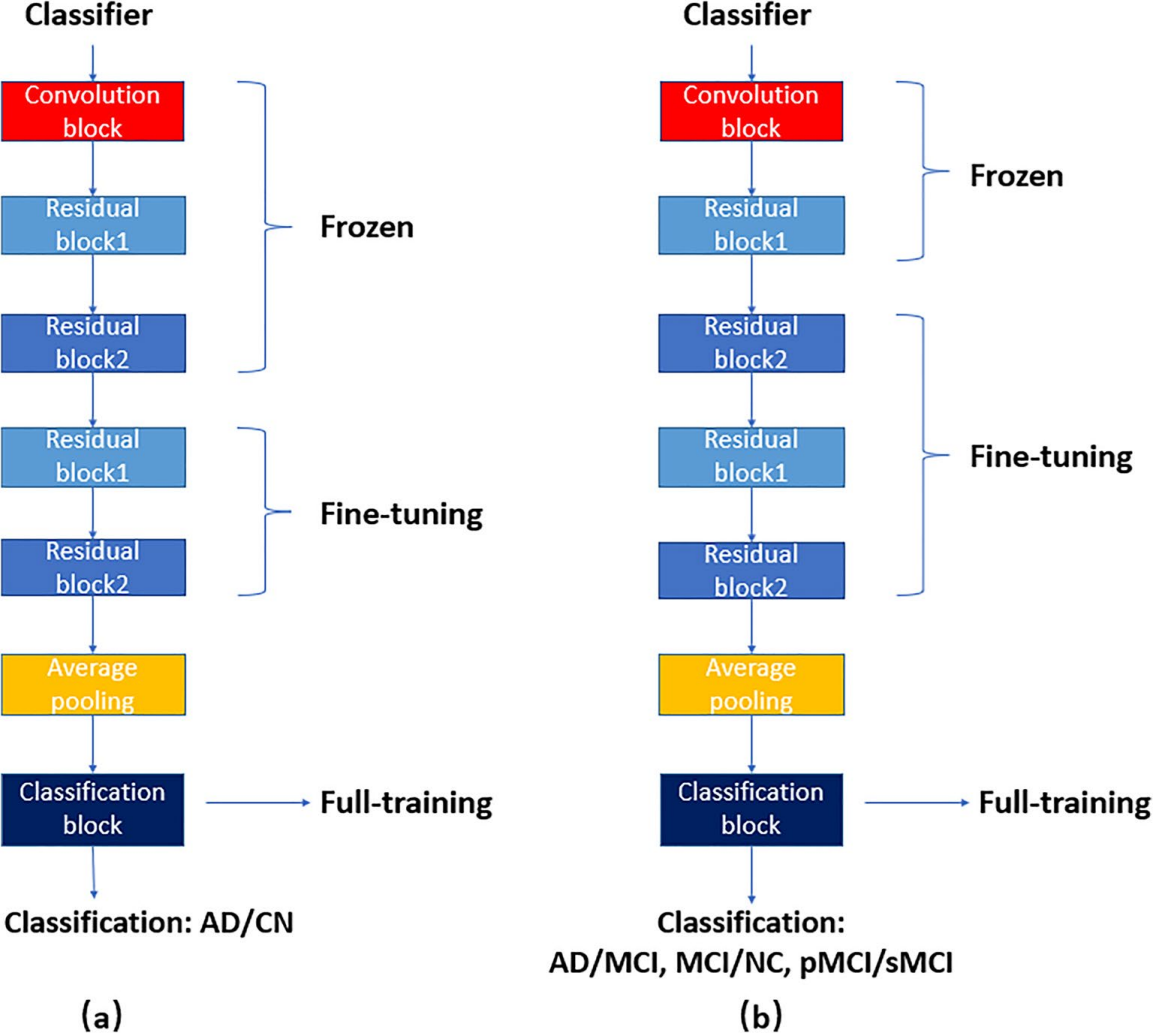
The network architecture of D-classifier.

As it has been shown in Fig. 1, the proposed 3D DCGAN was trained using the whole training set, including AD, MCI, and CN. The 3D CNN classifier (D-classifier) shares the same convolution architecture with D before the output layer, which can utilize the supplementary information learned in the training of 3D DCGAN. In order to achieve satisfactory classification performance in AD diagnosis, the procedure of transfer learning was adopted, and the output layer of the pre-trained D was changed to a classification block (three fully-connected layers). During the second-round, the parameters in the convolution block and the first two residual blocks were kept unchanged, while the weights of the rest of the residual blocks were fine-tuned, and the fully connected layers were fully trained. The architecture of the D-classifier in the AD versus CN classification task is shown in Fig. 3a.

In order to utilize the supplementary knowledge learned from the AD versus CN classification task, the third-round procedure was adopted, for which the D-classifier from the AD versus CN classification was transferred to the classifications of AD versus MCI, MCI versus CN, and MCI conversion prediction. During the third-round, the convolution block and the first residual block were frozen, residual blocks were fine-tuned, and the fully connected layers were fully trained. The architecture of the D-classifiers in MCI related binary classification tasks is shown in Fig. 3b.

### Mapping disease regions

Making the results more logical and explainable is crucial in many CNN applications related to medical imaging. Gradient-weighted class activation mapping (Grad-CAM) is a very powerful tool for providing an explainable visualization of CNN and could construct the visual clarification for any CNN to learn more about the model's decision-making process. It makes use of the gradient information of the target class, which flows into the last convolutional layer of the second residual block1 and contains the spatial information indicating discriminative regions for classifications. The class activation mapping is generated by concentrating on the specific portion of image discriminative features that the model used for classification. To illustrate the region of interest for CNN and carry out the quantitative analysis, we generated a heatmap by averaging the heatmaps with prediction probabilities larger than 0.7 for each class (real neuroimaging, AD, and CN classes).

### Performance metrics

To evaluate the performance of our classifiers, the following metrics were calculated: accuracy, sensitivity, precision, and AUC. These metrics are formulated as follows:

1 $$Accuracy=\frac{TP+TN}{TP+TN+FN+FP}$$

2 $$Sensitivity=\frac{TP}{TP+FN}$$

3 $$Precision=\frac{TP}{TP+FP}$$

where TP, FP, TN, and FN are the numbers of true positives, false positives, true negatives, and false negatives, respectively. AUC is calculated based on the area under the receiver operating characteristic curve.

### Ethical approval

The Ethics committees/institutional review boards that approved the ADNI study are: Albany Medical Center Committee on Research Involving Human Subjects Institutional Review Board, Boston University Medical Campus and Boston Medical Center Institutional Review Board, Butler Hospital Institutional Review Board, Cleveland Clinic Institutional Review Board, Columbia University Medical Center Institutional Review Board, Duke University Health System Institutional Review Board, Emory Institutional Review Board, Georgetown University Institutional Review Board, Health Sciences Institutional Review Board, Houston Methodist Institutional Review Board, Howard University Office of Regulatory Research Compliance, Icahn School of Medicine at Mount Sinai Program for the Protection of Human Subjects, Indiana University Institutional Review Board, Institutional Review Board of Baylor College of Medicine, Jewish General Hospital Research Ethics Board, Johns Hopkins Medicine Institutional Review Board, Lifespan—Rhode Island Hospital Institutional Review Board, Mayo Clinic Institutional Review Board, Mount Sinai Medical Center Institutional Review Board, Nathan Kline Institute for Psychiatric Research & Rockland Psychiatric Center Institutional Review Board, New York University Langone Medical Center School of Medicine Institutional Review Board, Northwestern University Institutional Review Board, Oregon Health and Science University Institutional Review Board, Partners Human Research Committee Research Ethics, Board Sunnybrook Health Sciences Centre, Roper St. Francis Healthcare Institutional Review Board, Rush University Medical Center Institutional Review Board, St. Joseph’s Phoenix Institutional Review Board, Stanford Institutional Review Board, The Ohio State University Institutional Review Board, University Hospitals Cleveland Medical Center Institutional Review Board, University of Alabama Office of the IRB, University of British Columbia Research Ethics Board, University of California Davis Institutional Review Board Administration, University of California Los Angeles Office of the Human Research Protection Program, University of California San Diego Human Research Protections Program, University of California San Francisco Human Research Protection Program, University of Iowa Institutional Review Board, University of Kansas Medical Center Human Subjects Committee, University of Kentucky Medical Institutional Review Board, University of Michigan Medical School Institutional Review Board, University of Pennsylvania Institutional Review Board, University of Pittsburgh Institutional Review Board, University of Rochester Research Subjects Review Board, University of South Florida Institutional Review Board, University of Southern, California Institutional Review Board, UT Southwestern Institution Review Board, VA Long Beach Healthcare System Institutional Review Board, Vanderbilt University Medical Center Institutional Review Board, Wake Forest School of Medicine Institutional Review Board, Washington University School of Medicine Institutional Review Board, Western Institutional Review Board, Western University Health Sciences Research Ethics Board, and Yale University Institutional Review Board.

## Consent to participate

Informed consent was obtained from all participants included in the study.

## Experiment and results

### Experiment implementation

All models in this work were deployed in Python 3.7.9 and Keras in TensorFlow 2.4 packages on a workstation with an Intel Xeon W-2223 CPU with 16 GB of RAM and an NVIDIA GeForce RTX 3090 GPU with 24 GB.

**Figure 4:**
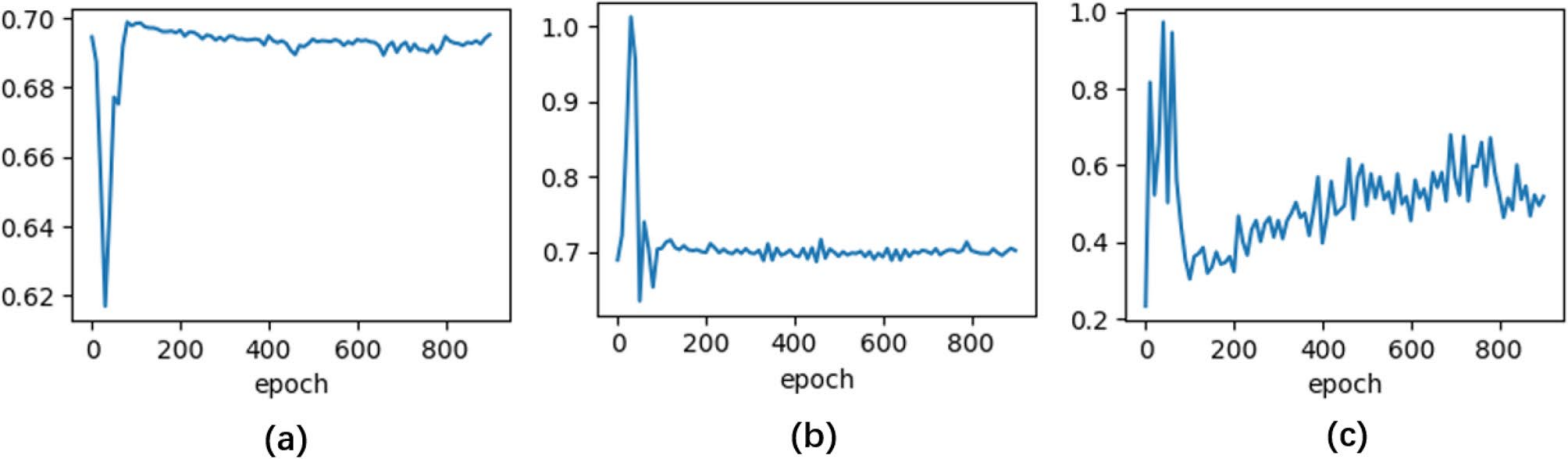
Training process of the proposed 3D DCGAN. (**a**) The loss of D (**b**) the loss of G (**c**) the output of D.

In the DCGAN model, binary cross entropy was used as a loss function. In the training phase, the batch size was set to 16, and the parameters were estimated through the Adam optimizer, with initial learning rates of 2 × 10^–3^ and 2 × 10^–4^ for the G and D, respectively. As training epochs increased, the losses of the validation set from both the G and the D converged to certain constant numbers, and the D’s accuracy hovered around 50%, indicating that the DCGAN had finally approached Nash equilibrium. The DCGAN was trained for 1000 epochs. Figure 4 demonstrates the loss and accuracy of G and D in the validation set. We adopted the procedure of transfer learning for binary classification; the 3D D-classifier was trained using the Adam optimizer with an initial learning rate of 1 × 10^–3^ to iteratively fine-tune the weights with the error back-propagation iterative algorithm. The loss of the classifier was computed with weighted binary cross entropy, and the weights were determined by the ratio of samples in the classes.

### Impact of the frozen layers

Transfer learning enabled us to skip weight recalculations and upgrades for frozen layers. Due to partial updates of convolutional layer parameters, fine-tuning after transfer learning is less expensive than learning from scratch. Thus, we look at the performance of transfer learning, varying the number of frozen layers. Generally, the first several blocks of the pre-trained D are good for capturing low-level features in the images, such as boundaries, edges, and shapes. The subsequent layers aim at capturing common features that sMRIs have. However, they are not fully effective for specific classification tasks, such as AD diagnosis. If a certain convolutional block of the D-classifier is finely tuned, it can perform more specialized for a particular classification task. In our experiments, we determined the optimal configuration by trial and error. The best performance was achieved when the Conv blocks were frozen up to residual block2, while the other layers were fine-tuned (Table 4).

**Table 4:** Classification performance of the pre-trained D-classifier with different frozen blocks.

Frozen blocks	Accuracy (%)	Sensitivity (%)	Precision (%)	AUC (%)
No frozen blocks	88.0	81.1	91.6	87.3
Convolution block	84.3	83.8	82.7	84.3
Convolution layer to residual block 1	84.3	94.6	76.4	85.3
Convolution layer to residual block 2	92.8	91.9	92.5	92.7
Convolution layer to residual block 3	88.0	86.5	86.5	87.8
Convolution layer to residual block 4	83.1	78.4	82.9	82.7

### Impact of model dimensions

The 3D CNN model can utilize all the information from the 3D sMRIs, while the 2D sliced images can only use some of the information. However, the number of kernels used in fully connected layers is significantly greater in 3D CNNs than in 2D CNNs, and the likelihood of overfitting increases. It is therefore important to assess whether a 2D or 3D model is more appropriate for classifying AD. We thus compared 2D and 3D DC-GAN models. Table 5 shows the overall performance of the proposed method. The proposed 3D DCGAN based model is better than a formerly proposed multi-slice 2D DCGAN based classifier^14^ that obtained accuracies of 90.4%, 74.6%, 69.1% and 66.7% for the diagnoses of AD versus CN, AD versus MCI, MCI versus CN, and pMCI versus sMCI, respectively. The proposed method shared the same populations and preprocessing steps as the previous 2D based work. The better performance is due to the use of 3D convolution operations, where the 3D model can better exploit the richer spatial and background 3D information in sMRI.

**Table 5:** Results of the proposed method in four binary classification tasks.

Tasks	Accuracy (%)	Sensitivity (%)	Precision (%)	AUC (%)
AD versus CN	92.8	91.9	92.5	92.7
AD versus MCI	78.1	54.1	71.3	71.8
MCI versus CN	76.4	81.8	81.4	74.6
pMCI versus sMCI	63.5	51.9	58.7	62.0

### Impact of training strategy

Increasing the convolution dimension from two to three leads to an increase in the number of parameters. The more parameters a 3D CNN must learn, the larger the training data set required to overcome the overfitting problem. To get beyond dataset constraints, training strategy advancements are required. Table 6 shows the results of classification accuracy for three 3D CNN architectures, with two models (VGG-like CNN and D-classifier-like CNN) trained from scratch and one model (D-classifier) using our three-round training procedure. The results demonstrated that the classifier trained with the three-round training procedure yielded better results than those 3D CNNs trained from scratch. An interesting finding was that the simple architecture (VGG-like CNN) led to better testing results, compared to the more complex architecture (residual network based D-classifier-like CNN), as it is less prone to overfitting when trained from scratch. During the training of DCGAN, D focuses on image discrimination and guides G, which focuses on image generation, to create images that have similar visual and statistical features to the training set. Both networks try to learn deep representations from high-dimensional distributions of sMRI data. The combination of unsupervised sMRI feature learning and feature transfer can boost image classification performance with small to medium-sized training samples.

**Table 6:** Classification performance of three classifiers.

Methods	Accuracy (%)		
	AD versus CN	AD versus MCI	MCI versus CN
VGG-like CNN	85.5	65.8	74.0
D-classifier-like CNN	84.3	71.9	69.9
D-classifier	92.8	78.1	76.4

### Impact of sample size

We also investigated the impact of sample size on classification performance. When keeping the same model design and reducing the training data to two-thirds or one-half of the original size and keeping the test sample size the same, the 3D classifier was tested to see whether an insufficient training set led to overfitting (Table 7). Reducing the training sample size to one-half of the original samples had a relatively small impact on accuracy for 3D CNNs trained from scratch, with a drop of 4.2% and 1.4% for VGG-like and D-classifier-like 3D CNNs, respectively. It is also observed that reduction of the training set has a significant negative effect on the D-classifier, with half of the training data decreasing the accuracy by 9.2%. The D-classifier outperforms other models regardless of the number of samples, and this advantage becomes more and more obvious as the amount of data increases. Taken together, the above results suggest that the proposed learning procedure in the D-classifier is more beneficial for training a robust model when the sample size is small.

**Table 7:** Classification performance of three models’ comparison with varying sample size for AD versus CN.

Models	Sample size 100%	Sample size 67%	Sample size 50%
VGG-like CNN	85.5	83.5	81.9
D-classifier-like CNN	84.3	84.3	83.1
D-classifier	92.8	88.0	84.3

### Model's generalizability

Using the ADNI-2 dataset, we further confirmed the model's generalizability. ADNI-2 and ADNI-1 (1.5 T SPGR and 3 T MPRAGE) can be regarded as separate studies due to major scanner updates. At ADNI-2 sites with 3 T MRI scanners, sMRI images were produced using a 3D MP-RAGE T1-weighted sequence. The test set consisted of baseline scans from 105 CN individuals and 187 AD patients. We have had success with the ADNI-2 dataset; the values for accuracy, sensitivity, and precision were 85.6%, 87.1%, and 90.1%, respectively. In other words, the model put forward in this study has the potential to be used in more extensive research.

### Model's interpretability

**Figure 5:**
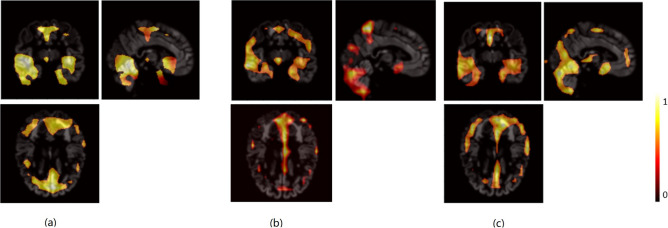
Visualization of features recognized by the trained CNN. (**a**) The average heatmap of the DCGAN training set participants in the D model. (**b**) The average heatmap of the AD patients from the test set in D-classifier, and (**c**) the average heatmap of the CN participants from the test set in D-classifier.

In order to better understand the model and identify brain regions linked to classification, the 3D Grad-CAM approach was applied in this work. We generated average relevance heatmaps for each class after weight back-propagation of trained models. The heatmaps of intermediate slices from the coronal, cross-sectional, and sagittal planes are shown in Fig. 5. Figure 5a depicts the average heatmap of the DCGAN training set participants. The average heatmaps of the AD patients and CN participants in the AD versus CN classification from the test set are shown in Fig. 5b. As shown in Fig. 5, the heatmaps of real neuroimages obtained by the D model in unsupervised learning have a large degree of overlap in the activation regions when compared with the AD vs. CN heatmaps obtained by the D-classifier in the AD and CN classification tasks. This suggests that the unsupervised pre-training of DCGAN gives the AD classifier a good place to start, and that this will allow the model to converge more quickly and prevent overfitting than if it were trained from scratch. In addition, there are some differences between the heatmaps of the AD and CN groups, with the heatmaps of the AD group focusing more on brain areas known to be associated with AD, such as the inferior and middle temporal gyri, and the hippocampus.

## Discussion

When dealing with 3D sMRI, the simplest approach is to treat a 3D volume as a sequence of 2D slices, and apply a pretrained classic 2D CNN to them. However, this would lead to discontinuous predictions across slices. An effective way to avoid it is to expand the CNN from 2D to 3D. In comparison to a 2D CNN pre-trained on ImageNet, sMRI datasets normally are relatively small to train the 3D CNN from scratch. To address this challenge, we train a 3D CNN with a three-round learning procedure: unsupervised sMRI feature extraction followed by two rounds of transfer learning. It is proved by the above experimental results that our proposed method provides a solution by training the models with unsupervised adversarial learning and fine-tune the model for the target problem.

Significant research efforts have been made in recent years to build predictive models of AD dementia based on sMRI data using CNNs. The experimental results of some state-of-the-art approaches for staging the AD spectrum reported in the literature, and the proposed classifier in this paper are shown in Table 8. It includes one 2D CNN based method, and thirteen 3D CNN based approaches (i.e., patch-level, ROI-level, and subject level). It is worth noting that data leakage is a serious problem in AD research^9^, some papers published have used inessential additional information for AD diagnosis (e.g., data leakage)^25^. The articles that may have had data leakage were excluded from the comparison. Because the training was performed with a varying number of subjects, the results in Table 8 are not fully comparable. Overfitting is more likely to occur when the sample size is small. By roughly comparing the proposed approach with those studies, we obtained two observations. First, in contrast to those methods, our proposed approach was trained on a relatively small sample, which means the model has a relatively higher risk of overfitting. Nonetheless, three-round learning in 3D CNN provided comparable performance to those cutting-edge CNNs, demonstrating the effectiveness of the training procedure. Second, the proposed CNN was highly-performing in differentiating AD from CN, good-performing in differentiating AD from MCI, and MCI from CN, but relatively poor in predicting MCI conversion to AD within 24 months. Compared with AD classification, MCI conversion prediction presents a greater challenge, since structural atrophy caused by MCI may be insignificant. The DCGAN model generated images with similar characteristic features of sMRI, such as the anatomical configuration of GM in GMDM, which mimicked the morphology and anatomy of the brain. Due to the difficulties in 3D training, the synthetic sMRIs normally have a blurred anatomical structural boundary, and contain artificial texture. Unsupervised learning in DCGAN can only capture characteristics commonly shared among sMRI. More effort should be put into the design of GAN architecture in order to capture characteristics in subtle atrophies.

**Table 8:** Comparison of classification performance of state-of-art studies based on baseline sMRI data of ADNI.

Study	CNN model	Subject numbers (AD + CN)	Accuracy (%)			
			AD versus CN	AD versus MCI	MCI versus CN	pMCI versus sMCI
Cui et al., 2019^26^	3D subject-level	427	91.3	–	–	71.7
Hu et al., 2020^2,7^	3D subject-level	891	91.8	–	51.2	–
Huang et al., 2021^2,8^	3D subject-level	607	90.8	74.3	76.7	–
Kong et al., 2022^15^	3D subject-level	241	89.8	79.5	79.5	–
Li et al., 2019^16^	3D ROI-level	410	89.1		75	72.5
Li et al., 2021^17^	3D ROI-level	411	85.9		73.3	71
Lin et al., 2021^29^	3D ROI-level	595	89.3			72.9
Liu et al., 2018^30^	3D patch-level	856	91.1	–	–	76.9
Liu et al., 2020^18^	3D ROI-level	216	88.9	–	76.2	–
Pan et al., 2020^12^	2D slice-level	499	84	–	79	62
Shen et al., 2023^31^	3D patch-level	416	89.6	–	–	–
Wu et al., 2022^32^	3D subject-level	773	91.3	–	–	–
Zhang et al., 2021^33^	3D subject-level	743	93.5	80.8	81.6	–
Our approach	3D subject-level	416	92.8	78.1	76.4	63.5

The subject number represents the total subject numbers used in training, validation and testing.

### The subject number represents the total subject numbers used in training, validation and testing

Our findings should be viewed within the context of several limitations. First, DCGAN extracts informative characteristic features in an unsupervised manner from sMRI, and is capable of synthesizing sMRI from those features. GAN may have the ability to learn the complete distribution of sMRI when given a sufficiently large sample. During DCGAN training, we only used the images from the ADNI-1 dataset, which can't take full advantage of GAN’s unsupervised mechanism. In the future, more neuroimage datasets^34–36^, even those that do not contain AD subjects, can be added to DCGAN training, which offers a better sMRI feature representation capability accordingly. Second, CNNs are employed for both the G and D models. The extensions of the G and D networks were expected to help the DCGAN capture more relevant features from sMRI. Many advances have been proposed in CNN architectures: the residual block is used to increase network depth, the inception block is used to extract multi-scale features, the dense block is used to improve the direction of information flow, and the self-attention mechanism structure is capable of semantic feature extraction. Replacing the regular convolution layers in G and D with those innovative blocks or structures may improve the network’s ability to extract subtle anatomical features. However, DCGAN maintains the dynamic stability of the training between the G and the D. The better the D is, the more serious the gradient of the G disappears; the convergence of the cost functions may become unstable. Improved designs of GAN, such as least squares GAN (LSGAN)^37^, Wasserstein GAN (WGAN)^38^, and energy-based GAN (EBGAN)^39^ can be adopted to improve the model’s performance and avoid vanishing gradients and mode collapse.

## Conclusions

AD is recognized as an irreversible degenerative disease. Recently, deep learning methods, especially 3D CNN, have been used for AD classification in the field of neuroimaging with some success. Insufficient sample size and high-dimensional feature representations are the main challenges of 3D CNN^40^. In this paper, we propose a three-round learning procedure for a DCGAN-based classifier to address the issue of overfitting susceptibility of 3D models. To work on 3D sMRIs, we extend the original 2D DCGAN to 3D by redesigning the architecture; the structure of D was improved by introducing residual blocks to avoid the disappearance of gradient. After unsupervised training of the 3D DCGAN, a feature extractor D for extracting common features of sMRI is obtained, and 3D Grad-CAM shows that it provides a good starting point for AD classification. The two-stage supervised transfer learning strategy accelerated the learning process and extracted more meaningful classification features for AD staging. The heatmaps of the AD group focused more on brain regions known to be associated with AD. The experimental results show that the performance of the 3D CNN suffers when it is trained with a limited number of samples. When the training set is small, the model obtained by the three-round learning procedure has an advantage over the model trained from scratch, and this advantage becomes more and more obvious as the amount of data increases. By using a three-round learning strategy, the problem of overfitting in 3D model training can be alleviated to some extent. The designed model performs comparably to existing state-of-the-art approaches on small to medium-sized datasets, proving its effectiveness.

## Data Availability

Data used in the study were obtained from the Alzheimer's Disease Neuroimaging Initiative (ADNI), a publicly available database (https://adni.loni.usc.edu) with no accession number.
